# Altered social trajectories and risks of violence among young Syrian women seeking refuge in Turkey: a qualitative study

**DOI:** 10.1186/s12905-019-0710-9

**Published:** 2019-01-10

**Authors:** Alison Wringe, Ekua Yankah, Tania Parks, Omar Mohamed, Mohamad Saleh, Olivia Speed, Rebecca Hémono, Bridget Relyea, Mahad Ibrahim, Jaspal S. Sandhu, Jennifer Scott

**Affiliations:** 1 Department of Population Health, London School of Hygiene and Tropical Medicine, Keppel St, London, UK; 2 Women and Health Alliance International (WAHA), Paris, France; 3 University of New South Wales, Sydney, Australia; 4 Institut d’études politiques de Paris, Paris, France; 5 University of California Berkeley, Berkeley, California, USA; 6 Gobee Group, Oakland, California, USA; 7 Relief Society for Syrian Refugees, Izmir, Turkey; 8 Department of Obstetrics and Gynecology, Beth Israel Deaconess Medical Center, Boston, MA USA; 9 Harvard Medical School, Boston, MA USA

**Keywords:** Refugee, Gender-based violence, Syria, Turkey, Qualitative, Adolescent

## Abstract

**Background:**

There is limited evidence regarding the ways in which displacement disrupts social norms, expectations and trajectories for adolescent girls and young women and the resulting impacts on their risks of violence. This knowledge gap is especially marked with regards to Syrian adolescent girls and young women seeking refuge in Turkey. We explored risks of gender-based violence against Syrian adolescent girls and young women in Turkey and examined how these risks were shaped by their displacement.

**Methods:**

Data were collected in August 2016 in Izmir, Turkey through five sex-specific focus group discussions with Syrian adolescents and young people (aged 15–25 years) and two mixed gender focus group discussions with Syrian adults (18 years and older). Group discussions covered the issues facing Syrian adolescents and young women in Turkey, and how these were influenced by their displacement. Discussions in Arabic were audio-recorded, transcribed and translated into English. Data were coded inductively, and analysed thematically.

**Results:**

Syrian adolescent girls and young women expressed an increased sense of vulnerability to violence since their displacement. Due to financial strains and limited educational opportunities, they were often encouraged by parents to work or marry, both of which they perceived to increase the risks of violence. In contrast, some adults suggested that marriage could protect adolescent girls and young women from risks of violence associated with working. Being alone outside the home was viewed as risky by all participants due to pervasive sexual, verbal and physical harassment, aggression, and even kidnapping attempts. To limit these risks, many parents reported keeping adolescent girls and young women at home, or ensuring that they were accompanied by male relatives when in public.

**Conclusions:**

Syrian adolescent girls and young women face multiple risks of violence following displacement related to altered social trajectories. Some family-based strategies to protect young women from violence could reinforce restrictive gender norms and increase risks of violence. Interventions to address violence should include providing safe spaces, access to education and safe transport for young women, and financial support for families as well as community-based interventions to address the daily risks of sexual harassment in public spaces.

## Background

Since 2011, the conflict in Syria has resulted in widespread displacement of the population, both internally, and to neighbouring countries and beyond [1, 2]. By the end of 2017, Turkey was home to an estimated 3.4 million Syrian refugees, of whom approximately one fifth were young women [1]. While displacement presents a myriad of challenges to refugees, adolescent girls and young women in particular are at increased risk of gender-based violence. Evidence from refugee contexts suggests that one in five displaced women experience some form of gender-based violence, including sexual harassment, sexual assault, and early marriage [3, 4].

The underlying causes of violence against adolescent girls and young women following displacement are multifaceted and vary across contexts. Structural factors shape young women’s risk of violence, including patriarchal values and gender norms that favourise, rationalise, and justify men’s domination and control over women in many cultures [5]. In the context of displacement, the breakdown of the rule of law and the collapse of family and community structures, reinforce social norms that condone violence against women [6]. Furthermore, in some settings, societal disregard for younger populations compounds these vulnerabilities, putting adolescent girls and young women at an even greater risk of violence [7].

Displacement can also increase adolescent girls and young women’s vulnerability to violence through changes to the social context and opportunities for young women who are transitioning from adolescence to adulthood. Interruptions to schooling may result from a lack of access to educational institutions in the host country, language barriers within schools, or financial burdens obligating young women to support their families economically, either through employment or marriage [8]. Parents may also perceive marriage to be a protective measure for their daughters in times of economic and social uncertainty [9], despite the specific risks of violence that earlier marriage entails, notably where cultural norms of deference to men may be reinforced through threats or acts of coercion and violence [10].

While it is recognised that adolescent girls and young women face high risks of violence in humanitarian and conflict settings, investigating the ways in which they manifest is challenging because of the rapid changes that often accompany humanitarian crises, particularly those involving widespread displacement [11]. In the context of the Syrian refugee crisis, this helps to explain the limited body of research on gender-based violence, with only a few studies investigating the role of the conflict in perpetuating violence against women in Syria [12], or describing the types of violence that have been experienced by displaced women during their passages to Europe [13]. While some studies have highlighted a growing frequency of early marriage among young Syrian refugees [14–16], there is an absence of empirical data on the ways in which displacement disrupts social norms, expectations and trajectories for adolescent girls and young women more broadly, and the resulting impacts on their risks of violence. This knowledge gap is especially marked with regards to Syrian adolescent girls and young women seeking refuge in Turkey, limiting the evidence base that is needed to develop interventions to address these risks.

We conducted a study that aimed to explore the relationship between displacement and risks of gender-based violence among Syrian adolescent girls and young women in Izmir, Turkey, and the feasibility of using mobile phone interventions to address risks of violence in this context. This manuscript draws on the data generated through this study to explore the interplay between the disruptions to social trajectories and risks of violence towards Syrian adolescent girls and young women in the context of their displacement to Izmir, Turkey.

## Methods

### Study setting

The study was conducted in August 2016 in the Basmane neighbourhood of Izmir, Turkey where two non-governmental organisations *Women and Health Alliance International* and *Relief Society of Syrian Refugees* collaboratively managed a community centre for Syrian refugees that offered Turkish language and cultural classes, psychological support, social services, referrals for legal services and women only “safe spaces”.

Izmir is the third most populous city in Turkey after Istanbul and Ankara. The coastline of Izmir is located eight kilometers from Greece, making it one of the largest transit points for refugees crossing over to Europe. Approximately 850,000 refugees, of whom around half were Syrian, transited through Izmir in 2015 [17]. At the time of the study, approximately 150,000 Syrian refugees were living in Izmir, with the majority unregistered and living in informal settlements [18]. Living conditions for Syrian refugees in Izmir are often cramped with several families typically residing together in rented accommodation in the poorest areas of the city’s suburbs. Syrian refugees in Izmir face widespread lack of access to social services including health and education beyond those provided by non-government organisations and formal employment opportunities are rare, leading to limited monthly disposable incomes [18].

### Study procedures

We conducted five sex-specific focus group discussions with young men and young women aged 15–25 years old and two with adult participants (mixed sex) aged 18 years and older. Women and men aged 15 years and older, of Syrian origin, and residing in Izmir for at least six months were eligible to participate in the study. Eligible participants were purposively sampled from those attending the community centre on fieldwork days, as well as through the refugee community networks of the organisations who were running the centre, to ensure a mix of ages, and marital statuses across both sexes (Table 1). Sampled participants were given information about the study and invited to participate in the discussion group that corresponded to their age and sex, held at the community centre on the same day. There were no refusals to participate among those invited.

**Table 1:** Composition of focus group discussions

Focus group discussion	Sex of participants (Female/Male)	Age range of participants (years)	Marital status of participants
Group #1: Young men (*n* = 5)	Male	17–23	Single (5)
Group #2: Young women (*n* = 4)	Female	16–17	Single (4)
Group #3: Community members (*n* = 4)	Female (3) Male (1)	20–49	Single (1) Divorced (1) Married (2)
Group #4: Young women (*n* = 3)	Female	17–39	Single (1) Divorced (1) Widowed (1)
Group #5: Community members (*n* = 5)	Female (2) Male (3)	23–38	Single (1) Married (3) Widowed (1)
Group #6: Young men (*n* = 4)	Male	16–23	Single (3) Married (1)
Group #7: Young women (*n* = 4)	Female	15–25	Single (1) Married (1) Divorced (1)

Following verbal informed consent, trained facilitators who were not previously known to participants, led discussions in Arabic. The sex and age of the facilitators matched the groups. Focus group discussions lasted approximately 60–90 min and were audio-recorded with the permission of the participants. The sessions included a vignette, a qualitative research tool in the form of a short story about a hypothetical person which can promote dialogue about sensitive topics or issues [19]. The facilitator read the vignette out loud, recounting a short story about a Syrian adolescent girl in Turkey whose family is experiencing financial difficulties (Table 2). The facilitator then asked for participants’ views about the scenario and how the family might respond to these challenges, using their responses to prompt further discussion of the issues facing young Syrian women in Turkey, and how these were influenced by their displacement. The discussion also covered risks, types and causes of violence in the community for Syrian women and girls, whether these had changed following their departure from Syria and existing support services. Additional topic areas for the focus group discussion with adults included their views on marriage and relationships, safety and security for women and girls, and mechanisms to protect themselves and their family from violence.

**Table 2:** Vignette used in the focus group discussion

Sana is a 15-year-old Syrian girl. She fled her village in rural Syria in December 2015 and is now living in Turkey with her parents, 16- and 13-year-old brothers and 10-year-old sister. Sana’s father was injured in Syria and hasn't been able to work since they arrived in Turkey. Her mother cleans houses for a Turkish family, but they are still struggling for money and are not sure how they are going to be able to pay this month’s rent. Until now, Sana has been going a school for Syrian students in Turkey. She enjoys learning but her parents say that she may have to stop soon if their money problems continue.	

Audio-recorded discussions were transcribed verbatim and translated into English. A thematic analysis was conducted whereby the transcripts and notes were first coded using an inductive approach to build up a coding framework. Codes were then reviewed to identify emerging themes and explore the relationships between them, drawing on feminist perspectives on the role of underlying structural and social drivers of violence [5, 10]. Data from the groups with young women, young men and adult community members were analysed separately in order to capture different perspectives on risks of violence in the lives of young Syrian women in this context.

### Ethical considerations

Verbal informed consent was obtained given the varied literacy levels of the participants, and to minimize the risks associated with having a document that could link individuals to their participation in the study. For participants aged 15 years old, parental consent was not obtained due to the high rate of child marriage under age 18 and acknowledging that many families may have been separated as a result of displacement [9, 20, 21]. Non-requirement of parental consent in humanitarian contexts, including in cases of family separation, is increasingly accepted as an ethically appropriate practice in order to ensure broad representation in research [22]. All discussions were conducted in private rooms within the community centre. Psychosocial support was available on site for study participants if needed. All study procedures, including those related to verbal consent, were approved by the ethics committees at Izmir University, the London School of Hygiene and Tropical Medicine (#11547) and the Beth Israel Deaconess Medical Center at Harvard Medical School (#2016P-000202).

## Results

The data revealed that displacement led many families to experience financial strains which in turn altered the social trajectories of many Syrian adolescents and young women, exposing them to new risks of violence, as detailed in the following sections:

### Displacement and changing social trajectories

Many young women lamented the limited educational opportunities in the new setting, recalling their schooling in Syria, while others expressed hope that they would be able to continue to study in the future. Many were conscious that schooling was being replaced by earlier marriage in their new location, with this seen as a strategy for coping with the financial burden that was faced by families and which had been triggered by displacement:

*No, when we were in Syria it wasn’t like this, everyone stayed in school. It was only when we came here and they saw the situation in Syria they started marrying off their daughters just so that they have less expenses.* (Group #2).

Marriage of Syrian adolescents and young women was not only seen as a practical means to reduce day-to-day expenses, but also as a way to secure access to a new source of income, if the future husband was wealthy:

*The offers that a young girl receives will be accepted by the parents if it could correspond to their financial needs. They do not care about the age or about the man himself, as long as he is wealthy.* (Group #6).

None of the young women approved of early marriage, and most adult women participants were equally resistant to the idea, sometimes citing it as a shameful act, although it was often acknowledged to be a viable option as a last resort. In contrast, most young men thought that it was acceptable for girls to get married at a young age in order to support the family financially or relieve some of the family’s financial burdens. Some young men went further by suggesting that young women should actively contribute to efforts to reduce a family’s financial burden by getting married:

*Someone needs to support the family financially. Even if her brother and father worked in Izmir I don’t think that it will be enough for the expenses.* (Group #5).

Entering the workforce was seen as an alternative means to secure income in the new setting, with several participants mentioning undertaking domestic or factory work. For many young women, finding employment to address their family’s financial concerns was more acceptable than marriage, as one young female participant reflected:

*Leaving school to [work to] help her parents is better than her getting married.* (Group #2).

### Risks of violence in relation to displacement and changing social trajectories

Most participants felt that adolescent girls and young women were far safer prior to their displacement, and that they were now exposed to new risks of violence, the most pervasive being verbal, sexual and physical street harassment when they were outside their homes. Young women described feelings of anxiety resulting from the generalized menacing environment with men on the streets, on corners, and in alleys drinking, smoking cannabis, and buying or taking pills and making threatening gestures, such as with knife blades. As one young woman described:

*Some guys hit blades on their wrists. The girl becomes spooked and traumatised from small things, as she doesn’t have a lot of strength.* (Group #2).

Although perpetrators of street harassment were reportedly from both the host and refugee community, some young women described how they were exposed to such behaviours for the first time, indicating that they found them contrary to the customs and norms in Syria. As one participant reported:

*Because they [young men] came here…and they learned different things and forgot the old. Half of them forgot how they were raised in Syria and they changed once they came here.* (Group #2).

Changed social trajectories resulting from displacement also exposed adolescent girls and young women to risks of violence. In the context of pervasive street harassment, travelling to a workplace alone was seen to incur a high risk of being verbally or physically aggressed, with one young woman reporting a kidnapping attempt:

*One time I was walking in the morning to work and a guy in his car yelled for me..…I told him [I’m] Syrian and he grabbed me and pulled me by the hand and was going to take me in his car.* (Group #2).

As a result of the risk of being harassed in the street, many women resorted to being accompanied by a male relative when they were outside the home, or travelling to work. One young woman explained the pervasive feeling of danger, except when accompanied by her father:

*There is no safety [here], we only feel safe when we are with our parents. If we are alone … someone will harass us on the street. Even with our mother we don’t feel safe and only feel safe with our father because he is a man. If they see a man with us they do not harass us, but if we were all women there is no safety at all.* (Group #2).

Although some young men expressed concern over the significant risks of street harassment that were involved with girls going to work in their new environment, they also suggested that it was a necessary sacrifice in light of the economic hardship associated with displacement. Adult women had additional concerns that working in private residences could expose adolescent girls and young women to abuse and sexual harassment from men in their employers’ houses. One woman stated:

*When the girl starts working, she will be prone to physical abuse, violence of course, sexual harassment, and many other things. No one knows what’s inside a home; it has secrets.* (Group #3).

Adolescent girls and young women reported that fewer educational opportunities resulted in spending more time at home, particularly for those who did not enter the workforce and did not get married. Even participants who supported the idea that young women should be economically productive suggested that the safest place for girls was at home. Some men explained how they sometimes restricted their daughters from going outside due to the worry that they would be exposed to street harassment:

*I am constantly worried about them, to be honest. I don’t allow them to go play outside the house. They play inside.* (Group #5).

However, being at home was seen to incur other risks, with both men and women referring to experiencing the “usual” sort of violence, perpetrated by parents, as a means to control the behavior of their daughters under certain circumstances:

*Man: I could beat my daughter if she is making mistakes, in order to put her back on the right track.* (Group # 6).

*Woman: Nobody beats for no reason. You could try to speak with her once, twice or even three times but after that it is done. You see that this human being is not willing to understand and the only way to make this stop is by violence.* (Group #6).

Although many participants referred to the financial benefits of marrying their daughters, marriage was also acknowledged to incur risks of intimate partner violence, which were exacerbated when girls got married whilst they were still “emotionally immature”. Furthermore, intimate partner violence was more likely to be condoned in the context of displacement, due to the changes in many families’ circumstances:

*Before, in Syria, if the husband beat his wife and she talked about it to her parents they would decide to bring her back home. But when you arrive to Turkey… your financial needs became different. The father of the girl turned out to be like: “Be patient dear, all men beat!” stuff like that…Beating women has become more acceptable… I mean you used to be scandalised by news concerning women being hit by their husbands…now...some people think that you have come to accept these things…because you are unstable and an immigrant.* (Group #6).

However, these concerns were sometimes balanced by the sense that marriage could protect adolescent girls and young women from being exposed to the risks of violence associated with working, or to more liberal attitudes towards having sexual relationships, or even getting drawn into prostitution:

*…and she could be helping out her family and drift to a different road (prostitution). This is very common at this age because they know how important money is. I know some very young girls who think of it this way: instead of working for 20 or 30 Lira per day, if they went down that bad road they could get 70 Lira in an hour or two. Then they tell themselves: why not?* (Group #6).

## Discussion

**Fig. 1:**
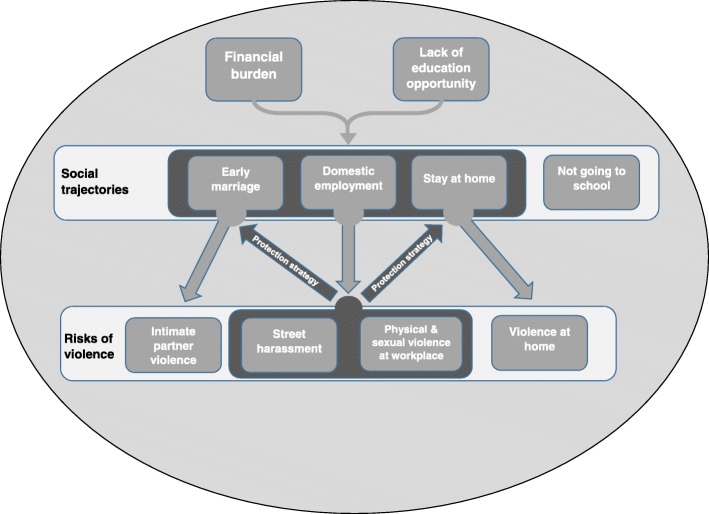
Conceptual framework showing the relationship between the social trajectories of Syrian adolescent girls and young women and risks of violence following displacement to Turkey

This qualitative study highlighted how displacement led to changes in social trajectories for Syrian adolescent girls and young women in Izmir, Turkey, and thereby exposed them to new risks of violence. Lost educational opportunities following displacement were often replaced by employment or marriage as means to achieve greater financial security. Increased risks of violence related to working included abuse from employers in the workplace, as well as the risks of verbal, physical, and sexual harassment whilst travelling to work. Some strategies adopted by families to protect adolescent girls and young women from violence, including keeping them at home, escorting them outside the home, or marrying them at an earlier age could reinforce gender inequitable norms, restrict their opportunities, and in some cases, increase their exposure to additional forms of violence (Fig. 1).

In our study, marriage for girls and young women was perceived to be both a risk factor for violence and a protective factor against violence, depending on the perspective. These findings should be considered in the context of other studies demonstrating that early marriage can have longstanding intergenerational impacts and affect women’s workforce participation, and that women who are married younger are less likely to have educational achievements [23, 24]. Similarly, displacement also caused older family members to impose limited mobility on adolescent girls and young women in an attempt to keep them safe at home, but in so doing, they could become economically vulnerable, disempowered, and more susceptible to different forms of violence, including physical violence from family members [25]. Other studies in refugee contexts have also noted how protection measures that are put in place to protect young girls and women from violence in an unfamiliar setting can ultimately serve to reinforce moralised social norms around “good” adolescent girl behavior that are restrictive in nature, and which can undermine gender equality [26].

The empirical link between the availability of economic resources, fewer educational opportunities and violence risks has also been documented across other settings [27], including among displaced populations [28, 29]. Our findings confirm the importance of addressing the underlying structural drivers of vulnerability to violence such as poverty, given the important role of economic concerns in terms of determining the social trajectories of Syrian adolescent girls and young women in this setting, and the consequences in terms of reinforcing restrictive gender norms. However, even in the absence of economic strife, it is likely educational opportunities would still be limited for young Syrian women in the new setting due to language barriers.

Our findings concur with the growing body of literature exploring how young peoples’ behaviours are shaped by social norms transmitted in the home by their parents and family members, as well as through their interactions with peers and other influential members of the community, particularly in relation to their sexual health and well-being [30]. As observed by Evans et al., we note that the choices that young people make are restricted by the moral, social, cultural and political boundaries that are enforced by the adults in their lives [31]. The agency of the adolescents and young women’ in our study was bounded through their triple placement within the lower echelons of the social hierarchies that were defined not only by their age, but also by their gender and their situation of displacement.

These results contribute to the growing body of evidence for interventions targeting adolescents and young people in conflict-affected contexts [7] and promoting resilience among youth living in high-threat environments [32]. In addition to interventions to address the structural drivers of violence in this setting, our findings also suggest that various practical measures could contribute to reducing the risks of violence faced by young Syrian women in this setting. This includes the provision of safe forms of transport, and community “safe spaces” which have been advocated by stakeholders involved in the Syrian refugee response, but which would also require meaningful parallel interventions to address the daily risks of sexual harassment in public spaces in order to be effective.

Our findings need to be considered in relation to study limitations including the potential for social desirability bias, which may influence participants’ responses when discussing sensitive topics such as violence and safety. As seen in other qualitative approaches using focus groups discussions, disclosure bias is a potential limitation in that responses may follow an accepted social narrative [33]. In addition, we sampled participants attending a community centre, and as such, we may not have captured views held by individuals who do not typically use these services. The use of a sampling frame covering all Syrian refugees in Izmir was not possible because many families were not officially registered in Turkey and did not have a fixed address. Finally, it is unclear to what extent some of the feelings of increased risks of violence among Syrian adolescent girls and young women were specific to this particular suburban setting, rather than to the effect of displacement per se, and thus caution should be exercised in extrapolating these findings to other settings.

## Conclusion

In conclusion, we found that Syrian adolescent girls and young women experienced physical, sexual and verbal harassment in their new environment. Displacement led to changes to their social trajectories, including entry into the workforce and earlier marriage, which consequently increased their exposure to violence. Some strategies to protect adolescents and young women from violence may reinforce restrictive gender norms and increase their risks of violence. Interventions to address violence against Syrian adolescent girls and young women in this setting should include financial support for young women and their families and greater access to education, as well as the provision of safe spaces and safe transport. Parallel community-based interventions should be implemented to address public sexual harassment.
